# Developing a lifestyle intervention program for overweight or obese preconception, pregnant and postpartum women using qualitative methods

**DOI:** 10.1038/s41598-022-06564-2

**Published:** 2022-02-15

**Authors:** Chee Wai Ku, Shu Hui Leow, Lay See Ong, Christina Erwin, Isabella Ong, Xiang Wen Ng, Jacinth J. X. Tan, Fabian Yap, Jerry Kok Yen Chan, See Ling Loy

**Affiliations:** 1 Duke-NUS Medical School, Singapore, 169857 Singapore; 2 Department of Reproductive Medicine, KK Women’s and Children’s Hospital, Singapore, 229899 Singapore; 3 School of Clinical Medicine, University of Cambridge, Cambridge, CB2 0SP UK; 4 Yong Loo Lin School of Medicine, National University of Singapore, Singapore, 119228 Singapore; 5 School of Social Sciences, Singapore Management University, Singapore, 178903 Singapore; 6 Department of Paediatrics, KK Women’s and Children’s Hospital, Singapore, 229899 Singapore; 7 Lee Kong Chian School of Medicine, Nanyang Technological University, Singapore, 636921 Singapore

**Keywords:** Reproductive disorders, Health services, Patient education, Public health, Weight management

## Abstract

The time period before, during and after pregnancy represents a unique opportunity for interventions to cultivate sustained healthy lifestyle behaviors to improve the metabolic health of mothers and their offspring. However, the success of a lifestyle intervention is dependent on uptake and continued compliance. To identify enablers and barriers towards engagement with a lifestyle intervention, thematic analysis of 15 in-depth interviews with overweight or obese women in the preconception, pregnancy or postpartum periods was undertaken, using the integrated-Promoting Action on Research Implementation in Health Services framework as a guide to systematically chart factors influencing adoption of a novel lifestyle intervention. Barrier factors include time constraints, poor baseline knowledge, family culture, food accessibility, and lack of relevant data sources. Enabling factors were motivation to be healthy for themselves and their offspring, family and social support, a holistic delivery platform providing desired information delivered at appropriate times, regular feedback, goal setting, and nudges. From the findings of this study, we propose components of an idealized lifestyle intervention including (i) taking a holistic life-course approach to education, (ii) using mobile health platforms to reduce barriers, provide personalized feedback and promote goal-setting, and (iii) health nudges to cultivate sustained lifestyle habits.

## Introduction

Overweight or obese women in the reproductive age group have an increased lifetime risk of adverse metabolic sequelae, along with subfertility, adverse obstetric outcomes in the mother and long-term adverse health outcomes for the child^1–9^. In line with the Developmental Origins of Health and Disease (DOHaD) paradigm, where early-life developmental influences are associated with later-life health and disease, optimizing the health of the mother can achieve the greatest benefit for mother and child^10^. Healthy diet and physical activity are key factors in addressing the obesity pandemic and related health complications^11^.

Various lifestyle interventions have been implemented in the preconception or pregnancy phases, which represent unique opportunities for cultivating sustained healthy lifestyle behaviors^12–16^. However, large randomized controlled studies of lifestyle interventions, such as dietary education, physical activity tracking, counselling workshops, and various combinations of these, have failed to achieve sustainable results^17,18^. In particular, lifestyle interventions initiated during pregnancy have been unsuccessful in preventing gestational diabetes or large for gestational age babies^19^. Few weight loss interventions have been conducted for women before conception^20^. While some have been shown to improve fertility, they did not result in significant improvements in metabolic outcomes^21–23^. Although there is considerable research and clear consensus regarding the goals of effective lifestyle interventions^20^, difficulty translating the evidence into clinical practice is widely documented. Key challenges to achieving outcomes in lifestyle interventions lie in successful engagement of participants and continued compliance with the intervention^20,24^. Enablers and barriers to behavioral change may arise from family and community characteristics, the local setting, and by social, environmental and policy contexts^13–16,25,26^. However, few studies have engaged with implementation science theories and integrated research findings to develop interventions that enable high engagement and sustained behavioral change. Hence, the optimal components of a lifestyle intervention for pregnancy that is acceptable, sustainable, and tailored to the concerns and needs of the target population needs to be determined.

The integrated-Promoting Action on Research Implementation in Health Services (i-PARIHS) framework supports successful implementation of evidence-based outcomes into real-world practice^27^. Specifically, it was designed to guide complex interventions in healthcare, providing a framework to systematically chart factors affecting implementation. By identifying barriers and enablers as perceived by users, it has previously been identified as a useful tool for iterative planning of the implementation of patient‐reported outcome measures (PROM) into routine oncology care^28^. As the eventual goal of this study is to create a lifestyle intervention program targeted at women with high body mass index (BMI), utilizing this framework will help to identify both enabler and barrier factors that should be resolved when designing a successful behavioral change intervention. Hence, the i-PARIHS framework was applied as a guide to understand how perceived enablers and barriers would influence the adoption of a lifestyle intervention program for the overweight or obese among preconception, pregnant and postnatal women, from which features of an ideal lifestyle intervention program with improved uptake and compliance are suggested.

## Methods

As the goal is to develop an intervention program that serves to accompany women throughout their preconception, pregnancy and postnatal journey, qualitative research was conducted as it provides medical researchers with deep insight into their patients’ life-worlds^29^. Participants were recruited from the general public and patients at KK Women’s and Children’s Hospital (KKH), Singapore, from clinical settings including pre- and post-natal clinics and the ward, as well as voluntary responses. A quota sampling method with a predetermined sample size was used to ensure that opinions surrounding all periods of preconception, pregnancy and postpartum were obtained. In total, 15 in-depth interviews were conducted at KKH between September 2020 to December 2020. Participants were females between 21 and 40 years of age with BMI ≥ 25 kg/m^2^. They were either in the preconception (n = 5), pregnancy (n = 5), or postnatal (n = 5) phases of their journey.

### Interview protocol

Data was gathered via two methods in each participant: by questionnaire and an in-depth interview session. Before the start of the interview session, participants filled out a short questionnaire that captured demographic data including age, ethnicity, BMI, smoking exposure, alcohol use, supplement use, educational attainment and employment status. One-to-one in-depth interviews were conducted due to the potentially sensitive topics surrounding obesity, weight and personal pregnancy experiences^30^.

A semi-structured interview guide was used during the in-depth interview session to provide systematic coverage of topics, allowing flexibility to explore the responses of participants when needed. This guide was developed based on a preliminary literature review into the knowledge, attitudes and beliefs regarding nutrition and lifestyle behavior among overweight and obese women before, during and after pregnancy^13,13–16,25,31–33^. The interview guide (Additional file 1) consisted of a standardized set of open-ended questions focusing on the knowledge, attitudes and perceptions of women towards a lifestyle intervention program, followed by further probing questions. These aimed to find out the perceived importance of their own and their offspring’s health; their baseline understanding of healthy behaviors, weight and health goals; motivators and barriers to achieving their goals; and acceptable dietary or other lifestyle interventions that they would be willing to undertake.

The in-depth interview sessions were conducted by three researchers who developed the interview guide. The interview sessions lasted around 60 min, were conducted in person using the English language, audio-recorded and transcribed verbatim. A team of research assistants transcribed the audio-recordings, which were mutually cross-checked for accuracy. Two independent researchers subsequently verified the transcripts for accuracy.

### Analysis

The in-depth interview transcripts were analyzed inductively using thematic analysis^34^. All transcripts were analyzed using NVivo (release 1.4.1, QSR International), a qualitative data analysis software^35^. Two study team members coded the transcripts independently. Subsequently, these codes were then discussed between the two study team members, resulting in identified themes. The development of codes and themes was an iterative and reflexive process where the team members shared their perspectives. Any discordances were resolved through consensus discussion. Themes generated were checked for data saturation, by ensuring that themes identified were elicited from the responses of multiple participants, to ensure an adequate sample size as it was unlikely that new information would be obtained from recruiting additional participants^36^.

The identified themes were subsequently mapped onto the i-PARIHS framework, which has four key constructs at its core: *Recipient*, *Context*, *Innovation* and *Facilitation*. These constructs were identified to be important considerations for a successful implementation of research findings^37^. The *Recipient* construct refers to a person’s characteristics that may affect implementation of the intervention^27^. The *Context* construct refers to non-people factors that can act to enable or constrain implementation, such as culture, leadership, the wider health system or organization^27^. Within this, we delved into how manipulating the environment surrounding a potential user may influence engagement with an intervention. The *Innovation* construct refers to the circumstantial interpretation of how the evidence should be applied to practice, which often involves tailoring to *Recipient* and *Context* factors^27^. The *Facilitation* construct refers to implementation processes that comprises of a facilitator (role) applying a process (a set of strategies and actions)^27^. As the facilitators apply the innovation on the recipients in the given context, each of the other constructs interact with the *Facilitation* construct.

Initial mapping was done independently by two study team members with discordance resolved through consensus discussions with a third study team member. Across the four i-PARIHS constructs, several barrier and enabler factors were identified. The finalized thematic map and its codes can be found in Additional file 2.

### Ethics approval and consent to participate

The current study was reviewed and approved by the SingHealth Centralized Institutional Review Board (CIRB Ref: 2020/2530). All participants signed an informed consent form before participating in the study. Identifying information was removed from the data prior to the analysis and all methods were performed in accordance with the relevant guidelines and regulations.

### Consent for publication

The authors declare that they provide consent for publication.

## Results

### Characteristics of the participants

Table 1 shows the participants’ baseline characteristics. Of the 15 participants, majority were Chinese, had completed tertiary education, were employed full-time, did not smoke, rarely or were non-alcohol drinkers, and consumed health supplements. They had a mean age of 33.8 ± 5.1 years and BMI of 33.7 ± 5.7 kg/m^2^.

**Table 1:** Characteristics of study participants.

Demographic	Total (n = 15)
**Age**	
< 30 years	3 (20%)
30–34 years	6 (40%)
≥ 35 years	6 (40%)
**Ethnicity**	
Chinese	9 (60%)
Malay	2 (13%)
Indian	4 (27%)
**Highest level of education**	
Secondary education	5 (33%)
Tertiary education	10 (67%)
**Employment**	
Not employed	4 (27%)
Part-time employed	1 (7%)
Full-time employed	10 (67%)
**Smoking status**	
Never	11 (73%)
Have stopped smoking	3 (20%)
Currently smoking	1 (7%)
**Alcohol intake**	
Never or less than 1 per month	12 (80%)
1–3 times per month	3 (20%)
**Supplement intake**	
No	3 (20%)
Yes	12 (80%)

### Qualitative findings

Ten basic themes representing participants’ perceived barriers and enablers to program participation and engagement were identified. They were organized into four main themes according to the constructs in the i-PARIHS framework, namely *Recipient*, *Context*, *Innovation* and *Facilitation*.

### Perceived barriers

#### *Recipient*: time constraints

Participants indicated that time pressure was a major challenge preventing them from engaging in healthy behaviors. They explained that the multiple roles and duties in their lives restrict them from having time to adopt healthy lifestyle habits such as exercising. *“As a mother, as a housewife, as a daughter-in-law, and as a person, I need to find time to do it all, to do everything. That is going to be very challenging”* (Interviewee 02)*.* Another participant said: *“Taking care of the kids and working, we really (sic) didn’t have time to exercise”* (Interviewee 06)*.*

#### *Recipient*: lack of knowledge about impact of obesity on health

Not all participants were aware of the health implications of being overweight and obese. *“Other than my weight, I don’t think there are any other health concerns”* (Interviewee 14)*.* Similar responses were given when participants were probed further on the potential health risks of obesity. “*Author: Do you have any thoughts about the impact of having a high BMI? Interviewee 02: Err no. Author: But specifically, do you know whether there are any negative effects? Interviewee 02: I don’t think so.”* Participants were also unaware about the implications of obesity on their offspring’s health. *“I feel that despite being overweight and obese, the risk to baby is minimal to none”* (Interviewee 01)*.*

#### *Context*: lack of relevant data sources

Participants are not able to access data sources relevant to their context. They pointed out that currently available information on guiding maternal healthy living, which are not relatable to the local culture, hinder them from making lifestyle changes. *“There are apps out there that track and tell you what to expect in pregnancy, what foods to eat, and what exercises to do, but they are only representative of western countries. I cannot find anything that is applicable to the local context so that’s why I’m not motivated to learn or follow the advice*” (Interviewee 07)*.* Some participants commented that existing sources are mostly contextualized in western cultural settings, which are not easily adopted in the local context nor appealing to Asian women. *“I think it’s because (these lifestyle apps are) are tailored to the angmoh*^ *context. Those apps are designed in western countries, (like) pumpkin soup (which) is not very Singaporean”* (Interviewee 08). (^Angmoh is a colloquial term referring to Caucasians, and may be used as an adjective that references western culture).

#### *Context*: family culture

Participants mentioned that the traditional mindset from elder family members plays a major part in determining their daily lifestyle practices. *“I think the old people are heavily influenced by their parents’ mindset. They tell me not to eat certain foods… Or to do certain things”* (Interviewee 04)*.* They expressed difficulty in communicating with their elderly in the family. *“My mother-in-law helped during my confinement period. We were totally lost. She assumed that what she did was the norm. But we have new products in the market now to help with childcare. She doesn’t know about them and she doesn’t believe in them. So it’s a bit tough with a bit of a clash, (and) my husband had to be the middleman”* (Interviewee 07). Eating together is a social practice in Asia^38^. During social gatherings and activities, individuals may adopt different eating behaviors that are not aligned with healthy diets*. “…when we are not meeting with our families, it’s probably easier to stick to the diet but when you meet during weekends, you’re happy, and there’s so much food, good food, to order in so we caved in many times”* (Interviewee 06)*.* In addition, family members living in the same household often share meals and lifestyle habits which are difficult to modify individually, separate from other household members. *“To be honest, because I don’t prepare my own food, I’m at the mercy of what my mom feels like cooking that day”* (Interviewee 09).

#### *Context*: food accessibility

Finally, there is a lack of an enabling environment to support positive lifestyle and dietary changes. Accessible and convenient unhealthy food options is a factor that perpetuates unhealthy diets. “because I’m late from work and I’m so hungry, because of the clinic schedule, we look at which (sic) shop doesn’t have a queue. Old Chang Kee* for example is the fastest, so its tempting to just buy (from there)” (Interviewee 13). (* Old Chang Kee is an established food retail chain in Singapore that sells deep fried snacks). This participant also added: *“…because every day when I come to work I’ll buy a lot of breakfast snacks, like from McDonalds probably (sic). So I just get one meal and sometimes I’ll order extra hash browns as well”* (Interviewee 13)*.*

### Perceived enablers

#### *Recipient*: motivation to change

Participants expressed motivation to make a healthy change, with the desire to be in better physical health for their offspring(s) when they grow up. “*Having a baby motivates (me) a lot, because I want to be there to see them grow up and I want to lose weight for my four children”* (Interviewee 02)*.* Another participant said: *“Yes, I can live for a long time (sic) to see my child get married (and) to have grandchildren”* (Interviewee 05). Participants were also motivated to change for their children. *“It’s my child who changed my diet. As my toddler joins me for meals, I (must) make sure the food is not fried, is not (sic) high in fat or seasoning, so I’m motivated by my child’s need(s) to eat healthily.”* (Interviewee 08).

#### *Context*: family and social support

Within the immediate environment, participants reported that family and social support would be a strong enabler. In particular, partner engagement with lifestyle changes alongside the prospective mother enabled mutual motivation. *“We were at our fittest together and we both encourage each other. So we both motivate each other”* (Interviewee 06). One participant highlighted the importance of engaging their partner in the program. *“(The program) should not just reach out to the mother, but also reach out to the father. (It) will be helpful if the partner, the father, also knows (the content of the program)”* (Interviewee 07). Support of the immediate family in the care of a young baby was also pivotal for new mothers to engage in healthy behaviors. *“My mother helps me to take care of the baby for about two hours, so that I can hit the gym or have a walk”* (Interviewee 04). Participants in our study also valued making social connections with peers to form a community of people going through similar experiences and provide mutual support. *“Mummies groups (sic) are good. Every pregnancy is different. Each baby is different. Everything is different. So with sharing by (sic) different mummies, it will give (me) confidence to hear that someone’s pregnancy (experience) is similar to mine. Then, I’m not the odd one out”* (Interviewee 07)*.* Another participant stated: *“Because there are things you cannot tell your parents, you cannot tell your other half. So telling people in the mummies group who understand you, then you will (sic) feel like, oh I’m not alone. And you do make good friends. Then (sic) you go for outings with the baby and learn from each other’s parenting. So I think it’s quite a good one”* (Interviewee 05)*.*

#### *Innovation*: desired and timely information

Participants indicated that concrete information about specific topics, such as healthy eating, exercise guide, mental health and breastfeeding support, should be included in the intervention program. *“I want to learn more about diet intake (sic), the type of exercises I can do, how to breastfeed, and also sleep training for the baby. I also want more assurance on the type of exercises that I can do, and for how long”* (Interviewee 08)*.* Another participant added: *“More on (the) breastfeeding issue … I still see (many) mummies struggling with breastfeeding”* (Interviewee 05)*.* Participants commented it was important that the information delivered was compatible with the specific needs and challenges faced at different phases of the journey from preconception to postpartum, necessitating different information, and intervention intensity. “*Before delivery you can provide (information) so they can be more well-equipped and at the same time, during the pregnancy, they can still go back to it to read up again”* (Interviewee 09). Participants were also keen to receive information about the postpartum period as early as preconception. *“Ideally, before I get pregnant, I want to learn about breastfeeding and sleep training. I only got in touch with breastfeeding and sleep training during pregnancy. I find that is too late and as a new mom, I don’t know how to analyze and filter all the information, so if there is some information on this during preconception, I can be mentally prepared for motherhood”* (Interviewee 08). The desired frequency and intensity of intervention was highlighted by a participant: *“(During) preconception, I would prefer to receive information every month, or every 2 months. As for health tips or nudges, I don’t mind receiving such information every day. During pregnancy, I would prefer receiving information every week. (In the) Postpartum period, I am happy to receive weekly newsletters”* (Interviewee 08)*.*

#### *Innovation*: holistic delivery platform

Participants wanted a single platform to deliver preconception, pregnancy and postnatal care which allows users to continue using the tool throughout the journey. *“There are apps online, but none are three-in-one—before pregnancy, pregnancy and postnatal, the whole journey. If you have an app that puts everything together, this app would be very widely used”* (Interviewee 05)*.* Most of the participants preferred a mobile application to deliver the intervention, with one of them saying*: “Mobile application, like an all-in-one application that I can refer to as and when I want to”* (Interviewee 11)*.* Another participant expressed a similar sentiment. *“Yes I will still prefer the app. Because you might be working. You might not have time to attend. I think the most efficient method is to have weekly classes. Because you need to know what to expect each week, what needs to be done. But no one will have time to attend a physical class every week. So I think an app is still better”* (Interviewee 08*)*. However, there were others who were hesitant to embrace new technology. *“I prefer physical classes that are face to face because I cannot concentrate when classes are online”* (Interviewee 15)*.*

#### *Facilitation*: frequent engagement

Some participants suggested the use of a monitoring logbook to monitor their health and feedback reports to guide healthy behaviors. *“Monitor our diet intake and exercise, that may keep us more engaged”* (Interviewee 11). This would help to provide ongoing motivation and enable them to troubleshoot their lifestyle. *“To improve engagement, you can have a monitoring logbook, get a gift or something to motivate (us)”* (Interviewee 02)*.* The desire for an interactive process was echoed by another participant*: “Yeah. Especially when there’s a section where you can actually (sic) ask a question, and get an answer, maybe not instantaneously but you know you may get one within the day, or maybe within a week, but there will be someone who answers the question. Or it could be become like a thumb’s up thing, where most voted questions get answered faster”* (Interviewee 09)*.* Participants also suggested nudges to support regular re-engagement, which would provide reminders and ongoing motivation to promote sustained behavior change. *“I feel like it would be good if there are notification pushes periodically maybe once or twice a week, but also if I were interested and I were to search it up within the app itself, its listed there as well. So, if I want to read forward, beyond those week by week pregnancy information pushes. For example what size (the baby bump) will be the following week, I can find that information (on the app) if I’m interested”* (Interviewee 09). Another participant added: *“Each of these nudges serves as a reminder when it’s time to run. Perhaps a nudge for the parents to think about whether they have any questions to ask the doctor”* (Interviewee 06)*.*

## Discussion

Ten enabler and barrier factors emerged from the interview process. These were framed through the lens of the i-PARIHS framework to understand how perceived barriers and enablers towards adoption of lifestyle changes shape participation and engagement with intervention programs. Among women of childbearing age who were overweight or obese, at different stages from preconception to postnatal, factors surrounding individual women, the sociocultural context, and key characteristics determining the uptake and continued efficacy of a lifestyle intervention were identified. Not tailoring the intervention sufficiently to these factors could explain the variable efficacy of existing interventions that aim to effect behavioral changes, such as diet modifications and increased physical activity^18,21,23,39–41^. Hence, we propose an intervention that mitigates these barriers and capitalizes on enabling factors identified in the results to ensure successful implementation and sustainable impact.

### Education across the life-course to address knowledge gaps

Despite strong motivation among mothers and prospective mothers to adopt healthier lifestyles for their babies, knowledge gaps and misconceptions are barriers that prevent the adoption of such behaviors^15,25,33^. Specifically, women are unaware that obesity before and during pregnancy has lifelong impact on maternal and child health. This may stem from insufficient guidance on healthy lifestyle behaviors applicable to the local context^13,16,25^. Addressing these through education will enable participants to perceive value in optimizing health for themselves and their children in the long-run, while providing practical suggestions that are culturally convenient, so women can act^42^. These form the considerations in designing an educational package as part of a lifestyle intervention program, aligned with our findings in this study.

First, the intervention should address the spectrum of needs and concerns beyond obesity and physical health, including physical activity and exercise, breastfeeding, and infant feeding practices. Participants of other studies have brought up similar concerns that are beyond the scope of the clinician’s agenda of targeting obesity or a specific health problem. Some topics of interest include support for miscarriage, sleep, mental health, postpartum care and baby care in the first year of life^43–45^. Notably, this indicates that participants desired a holistic intervention, which extends beyond their medical needs or a specific health concern to address their personal concerns as a woman and mother-to-be.

Second, the educational package should be relevant to users throughout the journey from preconception to postnatal phases. Information should be tailored to each phase as motivation and concerns for women differ at each of these phases. Before conceiving, the primary concern for prospective mothers is getting pregnant and optimizing fertility, hence information targeted at these topics are most likely to catch their interest. During pregnancy, women are concerned about how their lifestyle habits or symptoms affect the wellbeing of their fetus. Pregnancy-safe exercise plans at different trimesters are an example of information that can be provided. After delivery, women may worry about their recovery and care for their newborn, so topics such as breastfeeding and sleep training may be more relevant. In addition, women experience different time constraints during each phase. They have the least time postpartum as they are caring for their newborns, and the most time preconception where there are few medical visits and no fatigue from pregnancy. Thus, the intensity should also be tailored to these time constraints, with the greatest intensity before pregnancy, easing up during and after pregnancy.

There are many applications already available on the market, however these largely target a specific phase or aspect of the journey. Pregnant women currently use varying combinations of these apps^43,46^, but desire a comprehensive application that would serve all of their needs throughout the journey from preconception to postnatal. Taking a life-course approach achieves this by recognizing that an individual’s needs and concerns evolve over time, and tailors the intervention to suit their needs at different phases of the journey. In addition, initiating the intervention before conception gives women enough time to acquire nutrition knowledge and develop exercise habits^47^, enabling long-lasting lifestyle changes that are sustained before, during, and beyond pregnancy^20,47^. Continuing to support healthy behaviors in the postnatal period^45^ improves metabolic health when entering a subsequent pregnancy, and later in life.

Third, the identified barriers of contextual factors coupled with a lack of reliable locally relevant data sources show that difficulty integrating recommendations for lifestyle changes into local practices and habits prevent engagement with an intervention. Thus, the educational package must be designed with local practices, culture, and diet in mind. In particular, the influence of family culture on a person’s diet should be mitigated by engaging family members, especially partners, in the intervention program, which can be done by providing tips for exercising together or meal plans for the whole family. Not only does this promote healthy eating and lifestyle habits, but it also helps to establish spousal support throughout the journey of preconception, pregnancy and postpartum care. As it is a common practice for locals to purchase foods from food courts and food retailers (43), there is a need to incorporate education in identifying healthier food choices while eating out. This can integrate and augment current government policies and public health messaging such as the Healthier Dining Programme and Healthier Choice Symbol^48,49^.

## Promoting sustainable engagement with healthy lifestyle changes

The ultimate goal of lifestyle interventions is to cultivate sustained, long-term changes in behavior^20^. Strategies to enable these are a topic of interest and research^20,24^. Social support has been identified to be vital in promoting healthy behavior both in this study and others^43,46^. Meanwhile, time is a well-documented barrier to healthy lifestyle behaviors (e.g. exercising), particularly during the time surrounding pregnancy and motherhood^16,26,44,46^. Okesene-Gafa and colleagues (2016) found that perceived time constraint was cited as a likely reason for predicted non-participation if a nutritional intervention was created^16^. A longitudinal study conducted on Australian women showed that women’s perception of time pressure increased with number of children, as well as working hours they experienced^50^. Notably, majority of our participants are employed (73%) and thus our data provided a reflection of working women’s experiences and time constraints. For a lifestyle intervention to successfully enable behavioral change, time as a barrier needs to be sufficiently addressed.

Nudge interventions are increasingly used to influence health behavior to regularly re-engage users, and as promising adjuncts to educational interventions to counteract the adverse effects of decision biases that inhibit people from acting rationally^51,52^. Nudges can be delivered in the form of push notifications or short text messages, e.g. via social media that provide information or guidance, motivational messages, activity reminders, or promote interaction with the intervention^53^. This facilitates short and regular interactions with the lifestyle intervention, continuously re-engaging the user and providing consistent reminders for habit formation to drive long-term behavioral change^45^. Importantly, the intervention needs to be responsive to users’ needs. Incorporating built-in self-evaluation and feedback processes would enable users to support change independently, thereby enhancing self-efficacy and self-control^54^. It also allows personalization of healthy messages and nudges through goal-setting and feedback, which have previously been found to be more effective than non-customized messages in improving self-efficacy and dietary behaviors^55^.

Taken together, a sustainable intervention needs to incorporate a range of complex features providing guidance and education throughout the pregnancy life-course, while enabling a high degree of personalization to individual needs with feedback, goal setting and regular nudges. This is best achieved through a mobile health (mHealth) platform. mHealth is broadly defined as medical and public health practice supported by mobile devices such as smartphones^56^. The main advantage of using a mHealth platform compatible with smartphones is that it can be accessed anywhere at any time^46^. The intervention can be delivered through small but frequent interactions with the mobile app that can be completed in pockets of free time, which fits in well with the modern lifestyle of city-dwelling women, who have also expressed that they do not want to be given too much information at once^43^. Further, each interaction with the intervention can be tailored to the amount of available time, for instance completing short exercise sessions or consuming bite-sized information^43^, allowing changes to be flexibly incorporated into daily life.

## Strengths and limitations

A major strength of this study is the inclusion of the important phases in a woman’s life journey, from preconception to pregnancy and postpartum, and with recruitment from community and hospital settings. The codes and themes that arose from women in the preconception, pregnancy or postpartum phases were similar and is likely to represent women’s attitudes towards lifestyle interventions throughout these phases in general. Singapore is a multi-racial country, and this cultural diversity is reflected in our sample, which may be representative of most Asian countries. However, given the small sample size, the generalizability of our findings will need to be further studied. Nevertheless, these findings may represent enablers and barriers in other first-world regions with similar characteristics where women lead modern lifestyles, taking on multiple roles at home, work and socially, with widespread use of mobile phones, as similar enablers and barriers have been reported in studies in other such localities^14–16,24,25,31,39,40,45,50,53,57^.

However, this study only interrogates the perspectives of end-users, and does not take into consideration the perspective of other stakeholders. This study has identified women’s partners and families as potential enablers. However, this is not explored in our study and should be a topic of further research given their apparent influence on an individual’s lifestyle habits. In addition, the literature in implementation science research has established lifestyle interventions as multidisciplinary and multistakeholder efforts, with constraints and barriers from other perspectives such as healthcare professionals, organizations, managers, policy makers and other public bodies that would influence the success of the intervention aside from that of end-users^14,24,27^. However, there is a paucity of research that integrates the interplay of perspectives of multiple stakeholders in a lifestyle intervention, which should be a topic of further research.

The study is further limited by the role of the researchers as interviewers, which may have given rise to a degree of interviewer and response bias^58^. These were mitigated to some degree by clearly defining the tools and methods for collecting data to ensure responses were recorded and analyzed systematically^36^. The status of interviewers as healthcare professionals would predispose to response biases such as social desirability bias, which was mitigated to a degree through use of individual interviews^59^.

## Conclusion

Obesity caused by poor lifestyle behaviors, including dietary choices and lack of physical activity, has long-term adverse health effects in women and their children. The preconception, pregnancy and postpartum time period represents a unique opportunity for intervening to cultivate sustained healthy lifestyle behaviors. Although most pregnancies are planned and mothers are intrinsically driven to provide the best for their offspring, they may not be equipped with knowledge about preconception, pregnancy and postnatal care to put this into effect. The success of a lifestyle intervention is heavily dependent on uptake and requires continued compliance, which has been a challenge in previous studies. Using the i-PARIHS framework to explore the perceived enablers and barriers of adopting a lifestyle intervention program among preconception, pregnant and postpartum women who are overweight or obese, we propose components of an idealized lifestyle intervention to improve metabolic health. This includes a holistic education package taking a life-course approach to improve women’s baseline knowledge of health; using a mobile health platform to reduce barriers to adopting lifestyle changes, provide personalized feedback and goal-setting; and regular health nudges to cultivate sustained lifestyle habits.

## Supplementary Information

Supplementary Information 1.

Supplementary Information 2.

## Data Availability

Please contact the corresponding author for more information.
