# Supplementary material for: Developing a lifestyle intervention program for overweight or obese preconception, pregnant and postpartum women using qualitative methods
Source: Sci Rep. 2022 Feb 15;12:2511. doi: 10.1038/s41598-022-06564-2 (PMC8847557; doi:10.1038/s41598-022-06564-2)
Supplement: Supplementary file 1 — Supplementary Information 1. [file 41598_2022_6564_MOESM1_ESM.docx]

Qualitative in-depth interview guide for preconception, pregnant and postnatal women who are overweight and obese

| **Preferences to keep healthy** | |
| --- | --- |
| How do you view the importance of health to **you** before, during and after pregnancy? | Probe on health complications of being overweight and obese on the **mother**, at each stage.  Which stage do they think is the most important for them to take care of their health?  Do they perceive themselves as being at risk? If so, what risk? |
| How do you view the importance of your health to **your baby** before, during and after pregnancy? | Probe on health complications of being overweight and obese on their **baby**, at each stage.  Which stage of health has the most important impact on baby?  Do they perceive themselves as putting extra risk on baby? If so, what risk? |
| What are the things you **will do** to keep yourself and your baby healthy? | Probe on their eating behavior, activity levels, general beliefs on health (Healthy weight/ balanced diet/ sleep well/ physically active…etc.). |
| Did anyone talk to you about the **lifestyle habits** to prepare for a healthy pregnancy and after delivery? | Who? What does healthy lifestyle mean to you?  Probe on the amount (eat more/ less) and types of food. Any food you should/ shouldn’t eat, and why?  Probe on the activities (types, intensity, duration). |
| Did anyone talk to you about the amount of **weight** you should lose/ gain to prepare for a healthy pregnancy and after delivery? | Who? Probe on the weight monitoring and ideal weight loss/ gain. |
| Do you have any **goal** for yourself before, during and after pregnancy? | Probe on ideas of nutrition, body image, weight, and activity, in relation to self-identity and motherhood. |
| What do you think might be the **motivators** for you in achieving those goals? | Probe on the perceived benefits to their babies (baby health/ ease of labor…etc.) or themselves, the role of health care providers and families (partner/mother). |
| What do you think might be the **difficulties** in achieving those goals? | Possible barriers to change (biology, self-determination, environment, psychosocial, cultural belief, knowledge, clear guidelines). |
| What **type of activities** would you prefer to participate in to help keep you healthy before, during and after pregnancy? | Probe on the possible interventions that might be a part of the HELMS program (6P tool, education handout/ briefing, blood test, nudges, interaction approach, platform, location).  Are there any differences in the preferred interventions (types and mode of delivery) at different stages of pregnancy? |
