# Supplementary material for: Developing a lifestyle intervention program for overweight or obese preconception, pregnant and postpartum women using qualitative methods
Source: Sci Rep. 2022 Feb 15;12:2511. doi: 10.1038/s41598-022-06564-2 (PMC8847557; doi:10.1038/s41598-022-06564-2)
Supplement: Supplementary file 2 — Supplementary Information 2. [file 41598_2022_6564_MOESM2_ESM.docx]

Development of codes and themes

| **Basic codes** | **Basic themes** | **Main themes** |
| --- | --- | --- |
| Phase and obesity specific effects | Lack of knowledge about impact of obesity on health | **Recipients** |
| Diet and nutrition |  |  |
| Physical activity |  |  |
| BMI and weight gain |  |  |
| Healthcare professionals |  |  |
| Personal health |  |  |
| Mental health |  |  |
| Improve on physical limitations |  |  |
| Internal motivators | Motivation to change |  |
| External motivators |  |  |
| Lack of time | Time constraints |  |
| Work commitments |  |  |
| Take care of children and family |  |  |
|  |  |  |
| Diet and nutrition | Desired and timely information | **Innovation** |
| Physical activity |  |  |
| Breastfeeding |  |  |
| Weight management approach |  |  |
| Tailored to phase of journey |  |  |
| Frequency and format |  |  |
| Single platform | Holistic delivery platform |  |
| One-to-one coaching |  |  |
| Small group workshops |  |  |
| Virtual classes |  |  |
|  |  |  |
| Mobile applications | Lack of relevant data sources | **Context** |
| Internet |  |  |
| Word of mouth |  |  |
| Healthcare professionals |  |  |
| Role of spouse | Family and social support |  |
| Family influence |  |  |
| Support groups |  |  |
| Eating with family | Family culture |  |
| Asian culture and old wives’ tales |  |  |
| Eating out | Food accessibility |  |
| Food cravings |  |  |
|  |  |  |
| Logbook | Frequent engagement | **Facilitation** |
| Monitoring and feedback |  |  |
| Reminder |  |  |
| Sustainability |  |  |
| Frequently asked questions |  |  |
| Tips and advice |  |  |
